# Gut Microbiota, Mild Cognitive Impairment and Dementia: A Systematic Review

**DOI:** 10.3390/neurolint17100155

**Published:** 2025-09-28

**Authors:** Claudio Tana, Samanta Moffa, Marco Tana, Claudio Ucciferri, Livia Moffa

**Affiliations:** 1 Internal Medicine Unit, Eastern Hospital, ASL Taranto, 74121 Taranto, Italy; 2 Department of Pharmacy, G. D’Annunzio University of Chieti, 66100 Chieti, Italy; 3 Internal Medicine Unit, ASL Lanciano Vasto Chieti, 66100 Chieti, Italy; 4 Infectious Disease Clinic, ASL Lanciano Vasto Chieti, 66100 Chieti, Italy

**Keywords:** gut-brain axis, microbiota, dementia, Alzheimer’s disease, inflammation, probiotics, mild cognitive impairment

## Abstract

Background: Alterations of the gut microbiota have been increasingly implicated in the pathogenesis of dementia through mechanisms involving systemic inflammation, immune dysregulation, and gut–brain axis disruption. Clinical evidence, however, remains fragmented. Objectives: This systematic review aimed to characterize gut microbiota profiles in individuals with mild cognitive impairment (MCI) or Alzheimer’s dementia (AD), explore mechanistic associations with neurodegeneration, and evaluate the impact of microbiota-targeted interventions on cognitive outcomes. Methods: Following PRISMA 2020 guidelines and a registered protocol (PROSPERO CRD420251074832), PubMed/Medline was searched through May 2025. Eligible studies included randomized controlled trials (RCTs) and cohort and case–control studies assessing microbiota composition or interventions in participants with MCI or AD. Results: Twenty-one studies were included (1 RCT, 20 observational; sample size 22–302). Most used 16S rRNA sequencing; one used shotgun metagenomics. Across cohorts, MCI and AD patients consistently showed reduced short-chain fatty acid-producing bacteria (Faecalibacterium, Ruminococcaceae, Lachnospiraceae) and increased pro-inflammatory taxa (Escherichia/Shigella, Enterobacteriaceae, Bacteroides). Several studies reported reduced microbial diversity. Specific taxa, including Akkermansia muciniphila and Faecalibacterium, were associated with amyloid burden, hippocampal atrophy, and cognitive decline. Environmental and dietary factors influenced microbial composition and cognition. The RCT reported that probiotic supplementation improved inflammatory markers and BDNF levels, although changes in microbiota composition were inconsistent. Conclusions: Gut dysbiosis is strongly associated with cognitive impairment and markers of neurodegeneration. Modulation of the microbiota through diet and probiotics emerges as a promising avenue for dementia prevention and management, though robust longitudinal and interventional studies are needed to confirm causality and therapeutic efficacy.

## 1. Introduction

### 1.1. Definition and Prevalence of Dementia

Dementia encompasses a heterogeneous group of neurocognitive disorders characterized by a progressive and typically irreversible decline in cognitive performance, affecting individuals with previously preserved intellectual functioning [1]. The impairment is sufficiently severe to interfere with autonomy in daily living and social functioning. Among the most prevalent forms are Alzheimer’s disease (AD), dementia with Lewy bodies (DLB), frontotemporal dementia (FD), and vascular dementia (VD), each with distinct pathological substrates and clinical pictures. In recent years, the concept of Mild Cognitive Impairment (MCI) has gained prominence as a potential transitional state between normal aging and overt dementia. MCI is defined by subtle but measurable cognitive deficits, often without substantial functional impairment, though a significant proportion of individuals with MCI will progress to a diagnosable dementia syndrome over time [2].

According to current estimates, approximately 55.2 million individuals worldwide are living with dementia, with over 60% residing in low- and middle-income countries. Each year, around 10 million new cases are diagnosed globally [3].

AD accounts for an estimated 60–70% of all dementia cases, followed by DLB and FD (together approximately 20%), and VD, which accounts for about 20% of cases [3].

Prevalence is significantly higher among older adults, with a female predominance and high mortality rates. The incidence of dementia increases exponentially with age, reaching as high as 105 new cases per 1000 individuals per year among those aged 90 years and older, particularly in the case of Alzheimer’s disease [4].

### 1.2. Importance of Non-Neurological Factors in Neurodegenerative Diseases

In recent years, several non-neurological factors have emerged as important contributors to the onset and progression of neurodegenerative diseases. Among these, chronic inflammation, immune dysregulation, metabolic syndrome, and alterations in the gut microbiota appear to play significant roles in pathogenic mechanisms. Chronic inflammation, for instance, promotes the release of pro-inflammatory cytokines such as IL-6, IL-1β, and TNF-α, which negatively affect the clearance of pathological proteins associated with neurodegenerative disorders, including AD and PD [5].

In this context, metabolic syndrome is of particular interest, as it is associated with low-grade systemic inflammation, oxidative stress, and blood–brain barrier dysfunction, all of which may accelerate neurodegenerative processes [6]. Additionally, chronic psychosocial stress has been shown to modulate microglial activation, shifting microglia toward a pro-inflammatory state that promotes neuroinflammation and neuronal damage [7].

Moreover, psychosocial variables such as low educational attainment, social isolation, and chronic stress may act as cofactors, reducing cognitive reserve and exacerbating disease progression [8,9].

Collectively, these findings highlight the complex, multifactorial nature of neurodegeneration, in which biological, metabolic, and psychosocial factors converge to modulate both vulnerability and prognosis [8].

### 1.3. Emerging Role of the Gut–Brain Axis in Cognitive Health

Among the emerging systemic contributors, gut microbiota dysbiosis has gained significant attention as a potential modulator of neurodegeneration through its influence on the gut–brain axis [10].

Alterations in microbial composition can disrupt intestinal barrier integrity, modulate peripheral and central immune responses, and are strongly implicated in microglial activation and neuroinflammatory cascades [11]. These mechanisms provide a plausible link between gastrointestinal health and cognitive decline, positioning the gut microbiota as a critical interface in the pathophysiology of dementia [12]. Understanding this bidirectional communication may open novel avenues for early detection, prevention, and therapeutic modulation of neurodegenerative diseases, with a particular focus on AD and MCI [11].

### 1.4. Rationale and Aim of the Study

Recent studies suggest that gut microbiota alterations may contribute to the development and progression of dementia through mechanisms involving systemic inflammation, gut barrier dysfunction, and modulation of the gut–brain axis. AD and MCI were chosen as primary focuses because AD is the most prevalent dementia subtype and the best studied in relation to gut microbiota, while MCI represents a transitional state with a high risk of progression to dementia [11]. Moreover, microbiota-targeted interventions in these populations, such as probiotic supplementation, have been suggested to modulate microbiota composition and may confer cognitive benefits, while dietary modifications have been associated with reductions in systemic inflammation [11]. Despite growing interest, clinical evidence remains scattered and heterogeneous, with limited synthesis across human studies. This review was conducted to consolidate current knowledge from observational and interventional studies in humans, clarify microbiota-related pathophysiological processes, and explore emerging therapeutic approaches. The findings aim to inform future clinical research and support the development of microbiota-based strategies for dementia prevention and management.

## 2. Material and Methods

This systematic review was developed following a protocol registered in PROSPERO (CRD420251074832) and conducted in accordance with the PRISMA 2020 guidelines. The research question was structured using the PICO framework:

- Population (P): Adults (≥55 years, with a mean age between 65 and 75 years) including MCI and AD;
- Intervention (I): Evaluation of gut microbiota composition;
- Comparator (C): Normal controls (NC) or healthy control participants;
- Outcome (O): Measures of cognitive performance (e.g., MMSE, MoCA, ADAS-Cog), neurodegenerative biomarkers (e.g., amyloid-β, phosphorylated tau), microbial diversity indices, and taxonomic changes.

### 2.1. Review Aim and Scope

The review aimed to investigate the role of gut microbiota alterations in the development and progression of dementia, as well as to evaluate the effects of microbiota-targeting interventions on cognitive outcomes. Specific objectives included: (1) characterizing microbiota profiles in individuals with MCI or AD; (2) exploring mechanistic links between dysbiosis and neurodegeneration; and (3) assessing the clinical impact of interventions such as probiotics and diet.

### 2.2. Eligibility Criteria

We included peer-reviewed clinical studies (randomized controlled trials, cohort and case–control studies) involving participants diagnosed with MCI or AD. Studies were eligible if they assessed gut microbiota composition or implemented interventions aimed at modulating the microbiota. Relevant outcomes included cognitive performance, gut microbiota characteristics, and related biomarkers (e.g., inflammatory or metabolic markers). Only articles published in English were considered.

### 2.3. Search Strategy

A comprehensive literature search was conducted in PubMed/Medline, covering studies from database inception to May 2025. The search strategy included Boolean combinations of terms such as (microbiota OR microbiome OR “gut-brain axis”) AND (“dementia” OR “Alzheimer’s disease” OR “mild cognitive impairment”).

Case reports, narrative reviews, and systematic reviews were excluded from the main analysis but were screened for relevant background information and potentially eligible primary studies for discussion purposes.

Additionally, the reference lists of all included articles were manually screened to identify further relevant studies.

**Figure 1:**
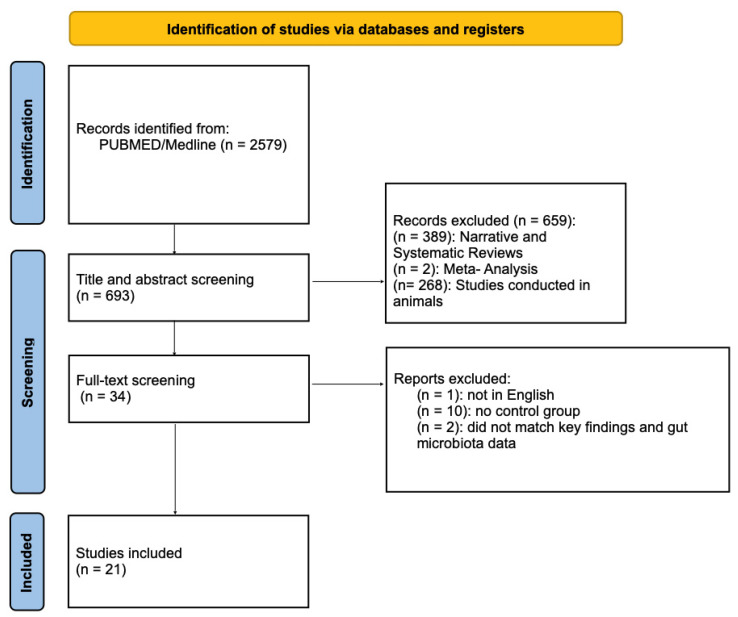
Shows the flowchart of the study selection process, including the number of records identified, screened, excluded, and ultimately included in the qualitative synthesis.

Figure 1 illustrates the study selection process according to the PRISMA flow diagram.

### 2.4. Study Selection and Data Extraction

Two reviewers independently (C.T. and L.M.) screened titles, abstracts, and full texts for inclusion. Data were extracted using a standardized form and included information on study design, population characteristics, microbiota analysis methods, cognitive assessments, and key results. Discrepancies were resolved through discussion or consultation with a third reviewer (S.M.).

### 2.5. Data Synthesis

Given the methodological diversity of the included studies, a qualitative synthesis was performed. Findings were grouped according to study design, type of cognitive impairment, microbiota findings, and presence or absence of microbiota-modifying interventions.

## 3. Results

A total of 21 studies exploring the relationship between gut microbiota and cognitive function in adults were included. The study selection process is in the PRISMA flow diagram (Figure 1). The designs comprised 1 randomized controlled trial and 20 cross-sectional observational studies, with sample sizes ranging from 22 to 302 participants. Populations included individuals with MCI and AD. The control group consisted of cognitively normal adults. Some of the included studies also reported participants with subjective cognitive decline (SCD). However, SCD was not used as a search keyword, inclusion criterion, or outcome of this review. It is presented only descriptively in Table 1, where applicable. Details on the bacterial taxa altered in MCI and AD across the 21 included studies are also presented in Table 1 [13,14,15,16,17,18,19,20,21,22,23,24,25,26,27,28,29,30,31,32,33]. This summarized the most frequent microbial changes and their association with cognitive outcomes.

Microbiota composition was predominantly assessed using 16s rRNA sequencing (in 20 studies), while one study [15] employed shotgun metagenomic sequencing. Cognitive function was evaluated with a range of tools, such as the Mini Mental State Examination (MMSE), Montreal Cognitive Assessment (MoCA), domain-specific cognitive tests (e.g., ADAS-Cog), neuroimaging findings (MRI, PET-imaging), and, in several studies, CSF biomarkers such as plasma amyloid-β and phosphorylated tau.

Across studies, a consistent association emerged between gut dysbiosis and cognitive impairment. RCTs demonstrated that probiotic supplementation was associated with increased Brain-Derived Neurotrophic Factor (BDNF) levels [13], reduced markers of systemic inflammation (e.g., IL-1B) and oxidative stress markers. However microbiota compositional changes were not reported in all RCTs. Table 2 illustrates the use of pre-, pro- and/or symbiotics and/or dietary modifications in patients with MCI and AD, emphasizing the changes in microbiota composition observed before and after the intervention and marker levels.

Observational data revealed recurrent alterations in gut microbial profiles of individuals with MCI and AD such as reduced abundance of short-chain fatty acid-producing bacteria (SCFA) including Faecalibacterium, Ruminococcaceae, Lachnospiraceae and Clostridiales in 10 studies. At the same time, 16 studies reported an increase in pro-inflammatory genera, such as Escherichia/Shigella, Enterobacteriaceae, and Bacteroides. Loss of microbial diversity, reduced alfa-diversity, was noted in 4 studies [17,28,30,32].

Several studies reported associations between specific microbial taxa and cognitive outcomes as well as Akkermansia muciniphila was inversely correlated with amyloid burden and cognitive decline [15,19]. The abundance of Faecalibacterium was lower in MCI and AD population across multiple cohorts and linked to reduced memory performance and hippocampal atrophy.

Environmental and lifestyle influences were also evident. Yuan et al. reported that exposure to airborne pollutants correlated with gut dysbiosis and cognitive impairment [28] while Saji et al. found that adherence to a traditional Japanese diet was associated with more favorable microbial profiles and a lower prevalence of dementia [20].

Other studies correlated specific genera to structural brain changes or biomarker profiles, such as increased free water in gray matter [18].

## 4. Discussion

This systematic review shows a consistent association between gut microbiota alterations and cognitive impairment, particularly in individuals with MCI and AD. Across the included studies, patients with cognitive decline exhibited a reduced abundance of SCFA-producing bacteria such as Faecalibacterium, Ruminococcaceae, and Lachnospiraceae [18,31,32], together with an increased prevalence of pro-inflammatory taxa including Escherichia/Shigella, Enterobacteriaceae, and Bacteroides [21,27,31]. These microbial shifts were frequently correlated with clinical and biological markers of neurodegeneration, including amyloid burden [15,24], hippocampal atrophy, systemic inflammation [19], and severity of cognitive decline.

Animal models of cognitive decline, particularly those mimicking AD, consistently support the involvement of the gut microbiota in the modulation of cognitive symptoms [10].

Several pathophysiological mechanisms may explain these associations. SCFAs are known to exert neuroprotective effects by maintaining blood–brain barrier integrity, modulating microglial activation, and regulating oxidative stress and neuronal metabolism [15,18]. Conversely, dysbiosis, characterized by an overrepresentation of pro-inflammatory bacteria such as Escherichia/Shigella, may contribute to chronic neuroinflammation and accelerate disease progression [22,28]. Specific taxa, such as Akkermansia muciniphila, have emerged as potential modulators, showing inverse associations with amyloid deposition and cognitive impairment, suggesting their possible role as protective biomarkers [15].

Environmental and lifestyle factors further modulate the gut–brain axis. Studies in Asian populations included in this review demonstrated that adherence to traditional dietary patterns, such as the Japanese diet [29] or high-quality anti-inflammatory diets [23], was associated with a more favorable microbial composition and reduced dementia prevalence. In contrast, diets rich in animal-based protein sources were associated with a less favorable microbial profile and an increased risk of cognitive decline [23].

Furthermore, the exposure to air pollutants such as fine particulate matter in rural China, correlated with gut dysbiosis, reduced plasma antioxidant enzymes and cognitive impairment [28]. Clinical characteristics also appear to play a role, as studies in obese African American cohorts showed that the association between gut dysbiosis and MCI was partly mediated by systemic inflammatory markers such as C-reactive protein (CRP) [19]. These findings reinforce the need to consider lifestyle and environmental determinants, along with genetic susceptibility factors such as APOE-ε4, when studying microbiota–dementia interactions.

Interventional evidence remains limited but provides encouraging signals. The only randomized controlled trial identified in this review [13] suggested that probiotic supplementation with Lactobacillus rhamnosus GG reduced Prevotella abundance and improved both cognitive outcomes and biological markers such as BDNF. Prebiotics, symbiotics, and dietary interventions are promising strategies, but robust evidence is still lacking.

### Limitation and Future Perspectives

Despite the growing interest in the role of the gut microbiota in neurodegenerative diseases [34,35], this review presents several limitations. Most included studies enrolled relatively small and heterogeneous cohorts, limiting the generalizability of findings. Methodological variability ranging from sequencing techniques (16S rRNA vs. shotgun metagenomics), cognitive assessment tools (MMSE, MoCA, biomarkers, neuroimaging), and outcome definitions further complicates cross-study comparisons. The predominance of cross-sectional designs also restricts causal inference. Some studies included only MCI patients, others compared cognitively healthy individuals with AD patients, and a few involved participants with SCD. In our review, SCD was considered only as a descriptive characteristic and not as a primary population, to maintain focus on MCI and AD. Moreover, inconsistent definitions of dysbiosis and heterogeneous clinical outcomes precluded a robust quantitative synthesis. This underscores the need for standardized diagnostic criteria, microbiota analysis techniques, and cognitive evaluation methods in future research.

Additional limitations include sex imbalances and insufficient attention to pharmacological or dietary factors. Few studies accounted for the influence of diet, baseline microbiota composition, or concomitant therapies such as probiotics, antibiotics, or other medications. Multimorbidity and polypharmacy in older adults may independently alter microbial composition and affect cognitive trajectories [36,37]. Commonly prescribed drugs such as proton pump inhibitors, antibiotics, metformin, and laxatives are known to modify gut microbial diversity, while cardiometabolic conditions including diabetes, hypertension, and obesity may act as mediators between dysbiosis and cognitive decline [38,39,40]. Lifestyle factors such as dietary patterns, physical activity, sleep quality, and alcohol consumption further complicate interpretation, as they affect both microbiota and brain health [41,42,43].

Reduced physical activity, in particular, contributes to sarcopenia, which has been consistently linked to cognitive decline through chronic inflammation, insulin resistance, and impaired protein metabolism [44,45]. Dysbiosis may exacerbate anabolic resistance by impairing energy balance and amino acid bioavailability, suggesting an emerging “gut–brain–muscle axis” that connects muscle deterioration with cognitive vulnerability [46,47,48]. Preliminary evidence also supports a beneficial role of probiotics in cognitive outcomes, especially when combined with regular physical activity. Such multimodal interventions may enhance microbial modulation, reduce inflammation, and promote neurotrophic signaling, offering promising strategies to preserve cognitive health [49,50].

Finally, limitations of the review process itself must be acknowledged. PubMed/Medline was the primary database used, although preliminary searches in Web of Science and Scopus did not identify additional eligible studies. Grey literature (e.g., theses, conference proceedings, trial registries) was excluded to ensure methodological rigor and restrict inclusion to peer-reviewed articles.

Future research should prioritize multicenter randomized trials with larger sample sizes, standardized methodologies, and extended follow-up periods to better establish causality and therapeutic potential [34]. Moreover, integrating advanced tools such as multi-omics profiling, machine learning, and artificial intelligence–driven predictive models could help disentangle the complex interactions within the gut–brain axis, identify patient subgroups most likely to benefit, and support the development of personalized nutritional and microbiota-based interventions for dementia prevention and treatment [35].

## 5. Conclusions

In summary, current evidence underscores the potential role of the gut microbiota as both a biomarker and a therapeutic target in cognitive decline. Dysbiosis appears not only to be associated with dementia but is also linked to structural brain changes and systemic inflammatory responses. Integrating microbiota research with neuroimaging, metabolomics, and immunology may pave the way for personalized approaches to dementia prevention and management. Ultimately, well-designed longitudinal and interventional studies will be critical to move from associative evidence toward causal understanding and clinical translation.

## Figures and Tables

**Table 1:** Gut Microbial Taxa Alterations in Patients with MCI and AD. This table provides an overview of the main studies investigating gut microbial taxa in patients with MCI and AD summarizing study design, analytical methods, and sample size. MCI: mild cognitive impairment; AD: Alzheimer’s Dementia, SCD: Subjective Cognitive Decline; NC: normal controls; MMSE: Mini-Mental State Examination; CDR: Clinical Dementia Rating; ADAS-cog; Alzheimer’s Disease Assessment Scale-Cognitive Subscale; RCPM: Raven’s Coloured Progressive Matrices; FAB: Frontal Assessment Battery; LM-WMSR: Logical Memory subtests I and II of the Wechsler Memory Scale-Revised; HAMD: Hamilton Depression Rating Scale; HAMA: Hamilton Anxiety Rating Scale; MoCA-B: Montreal Cognitive Assessment-Basic version; AVLT-D (long): Auditory Verbal Learning Test-long delayed recall; AVLT-R: Auditory Verbal Learning Test-recognition; AVLT-H: Auditory Verbal Learning Test- HuaShan version; STT-A: Shape Trails Test Part A, STT-B: Shape Trails Test Part B; AFT: Animal Fluency Test; BNT: Boston Naming Test; FAQ: Functional Activities Questionnaire; SCD-Q9: SCD Questionnaire 9, AD8: Ascertain Dementia questionnaire; IADL: Index for Activities of Daily Living.

Study	Type of Study	No. of Patients Included	Gender	Analysis	Cognitive Alteration	Cognitive Test	Altered Taxa	Dietary Issues	Treatment	*p* Value
Aljumaah et al., 2022 [13]	Randomized, double-blind, placebo-controlled trial	169 middle-aged (52–59 years) and older adults (60–75 years)	Placebo group: 28 M 55 F Probiotic group: 38 M 48 F	16S rRNA sequencing	MCI	MMSE	↑ Prevotella ruminicola, ↑ Bacteroides thetaiotaomicron, ↑ Dehalobacterium	Not reported	Daily supplementation with LGG	*p* = 0.0017
Li B. et al., 2019 [14]	Cross-sectional observational study	30 MCI 30 AD 30 NC	MCI 12 M AD 15 M NC 13 M	16S rRNA (fecal + blood)	MCI/AD	MMSE	↑ Escherichia spp. in both MCI/AD, reduced microbial diversity	Not reported	No intervention applied	*p* = 0.001
Fan et al., 2025 [15]	Observational cross-sectional study	439 individuals: 119 MCI 320 NC	MCI 51 M NC 111 M	Shotgun metagenomic sequencing (MetaPhlAn4, Illumina NovaSeq) of fecal samples	MCI	MMSE, CTT1, CTT2, Digit Span, DSST, Logical Memory (LMI/LMII), Semantic Verbal Fluency, Stroop, Boston Naming Test	↓ Akkermansia muciniphila; mixed Bacteroides, Ruminococcus species	Vegeterian → 19 Normal, 4 MCI Tea consuptionFrequently → 73 Normal, 24 MCI Coffee consumption → 122 Normal, 30 MCI	No intervention applied	*p* = 0.004
Wanapaisan et al., 2022 [16]	Cross-sectional observational	52 total: 20 NC 12 MCI 20 AD	NC 8 M MCI 6 M AD 10 M	16S rRNA sequencing of stool samples	MCI/AD	Diagnosis based on clinical, cognitive assessments (CDR, MMSE), plus brain imaging (MRI and amyloid PET)	↑ Escherichia–Shigella, Bacteroides, Holdemanella, Romboutsia, Megamonas; ↓ Faecalibacterium, Agathobacter, Clostridiales	AD group consumed significantly fewer vegetables than controls Rice was the most consumed carbohydrate across all groups	No intervention applied	*p* < 0.0001
Wang et al., 2024 [17]	Cross-sectional observational study	229 older adults: 74 MCI 131 NC	MCI 50 M NC 68 M	16S rRNA sequencing of fecal samples	MCI	MMSE classification	a-diversity lower in MCI vc NCI, ↑ Megamonas, Blautia, Pseudomonas, Stenotrophomonas, Veillonella	Not reported	No intervention applied	*p* < 0.001
Yamashiro et al., 2024 [18]	Cross-sectional observational	56 participants: 19 NC 19 MCI 18 AD	NC 7 M MCI 6 M AD 8 M	16S rRNA sequencing of fecal samples	MCI/AD	MMSE, MoCA, ADAS-Cog, CDR + free water MRI (FW)	↓ Anaerostipes, Lachnospiraceae UCG-004, [Ruminococcus] gnavus group	Not reported	No intervention applied	*p* = 0.003
McLeod et al., 2023 [19]	Case–control observational	60 obese, African American adults (55–76 yrs): 30 MCI 30 NC	NC 21 M MCI 21 M	16S rRNA sequencing of fecal samples	MCI	MoCA scores	↓ Parabacteroides distasonis; ↑ Dialister invisus, Streptococcus, Methanobrevibacter	Mediterranean diet: non-refined grains, potatoes, fruit, vegetables, legumes and nuts, fish, olive oil, alcohol, red meat and processed meat, poultry, and full-fat dairy products	No intervention applied	*p* = 0.04
Saji et al., 2019 [20]	Cross-sectional observational	82 Japanese older adults: 61 MCI 21 NC	MCI 28 M NC 11 M	16S rRNA sequencing of fecal samples	MCI	MMSE, CDR, ADAS-Cog, RCPM, FAB, LM-WMSR I, LM-WMSR II	↑ Bacteroides	Not reported	No intervention applied	*p =* 0.009
Zhu Z. et al., 2022 [21]	Cross-sectional observational	302 older adults: 94 NC 125 MCI 83 AD	NC 36 M MCI 49 M AD 30 M	16S rRNA sequencing of fecal samples	MCI/AD	CDR	↑ Erysipelatoclostridiaceae, Patescibacteria, Saccharimonadales; ↓ Alistipes, Faecalibacterium	Not reported	No intervention applied	*p <* 0.05
Kim E-J et al., 2023 [22]	Cross-sectional observational	80 elderly Koreans: 40 MCI 40 NC	MCI 6 M NC 2 M	16S rRNA sequencing of fecal samples	MCI	MoCA scores	↑ Bacteroides, Eubacterium nodatum group, Oribacterium, Rikenellaceae RC9; ↓ Prevotella, Coprococcus, Akkermansia	Not reported	No intervention applied	*p <* 0.05
Zhang X. et al., 2021 [23]	Case–control observational	127 participants: 75 MCI, 52 NC (Chinese)	MCI 36 M NC 24 M	16S rRNA sequencing of fecal samples	MCI	MoCA scores, MMSE	↑ Proteobacteria, Gammaproteobacteria; ↓ Faecalibacterium, Alistipes, Ruminococcaceae	Chinese Dietary Guidelines Index 2018: carbohydrates, grains and mixed beans, fruit, vegetables, soybean and nuts, meat and poultry, eggs, aquatic products, oil, salt, and alcohol	Diet quality	*p =* 0.008
Sheng C. et al., 2022 [24]	Cross-sectional observational	88 community-dwelling older adults: 34 CN– (Aβ−) 32 CN+ (Aβ+ preclinical AD) 22 CI (11 MCI, 11 AD)	CN- 8 M CN+ 10 M CI (MCI 8 M, AD 5 M)	16S rRNA sequencing of fecal samples	MCI/AD	AVLT, STT-A, STT-B, AFT, BNT, MoCA-B, FAQ, HAMD, HAMA	↑ Bacteroidetes; ↓ Firmicutes, Deltaproteobacteria, Desulfovibrionaceae, Faecalibacterium, Bilophila	Not reported	No intervention applied	*p =* 0.003
Sheng C. et al., 2021 [25]	Cross-sectional observational study	105 participants: 38 NC 53 SCD 14 CI (including MCI/AD)	NC 15 M SCD 10 M CI 4 M	16S rRNA sequencing of fecal samples	MCI/AD	SCD-Q9, AVLT-H, STT-A, STT-B, AFT, BNT, MoCA-B, FAQ, HAMD, HAMA	↓ Faecalibacterium, Clostridium	Not reported	No intervention applied	*p =* 0.021
Lwere K. et al., 2025 [26]	Cross-sectional observational	104 older adults in Uganda: 77 AD 14 MCI 13 NC (≥60 year)	AD 14 M MCI 3 M NC 4 M	16S rRNA sequencing of fecal samples	MCI/AD	MoCA, DSM--V/ICD--11 criteria	↑ Hafnia-Obesumbacterium, Dickeya; ↓ Novosphingobium, Staphylococcus	Not reported	No intervention applied	*p <* 0.05
Yıldırım S. et al., 2022 [27]	Cross-sectional observational	125 participants: 51 NC 27 MCI 47 AD Turkish cohort	NC 28 M MCI 16 M AD 24 M	16S rRNA amplicon sequencing (fecal)	AD	MMSE, CDR	↑ Bacteroides, Escherichia/Shigella, Subdoligranulum, Bilophila, Alistipes; ↓ Faecalibacterium, Lachnospiraceae, Ruminococcaceae	Not reported	No intervention applied	*p =* 0.04
Yuan et al., 2024 [28]	Cross-sectional observational	120 rural Chinese elderly: 81 AD 39 NC	AD 40 M NC 15 M	16S rRNA sequencing of fecal samples	AD	AD8 questionnaire	↑ Escherichia–Shigella, Akkermansia, Monoglobus; ↓ Faecalibacterium	Not reported	No intervention applied	*p =* 0.0235
Saji N. et al., 2022 [29]	Cross-sectional study	85 participants: 23 AD 42 MCI 20 NC	33 M	16S rRNA sequencing of fecal samples	AD	MMSE, CDR	↑ Faecalibacterium, Ruminococcus, Gemmiger; ↓ Bifidobacterium, Actinomyces, Parabacteroides	Japanese Diet: rice, miso, fish and shellfish, green and yellow vegetables, seaweed, pickles, fruit, soybeans and soybeanderived foods, mushrooms, beef and pork, chicken, green tea, and coffee	Adherence to Japanese diet	*p =* 0.006
Teigen L.M. et al., 2024 [30]	Cross-sectional observational	27 MCI. 11 iRBD, 39 cohabitant controls, 19 not cohabitants unrelated healthy controls	iRBD 9 M Cohabitant Controls 5 M	16S rRNA sequencing of fecal samples	MCI	MoCA	↑ Blautia, Collinsella; ↓ Bacteroides, Alistipes, Faecalibacterium, Lachnospiraceae	Not reported	No intervention applied	*p <* 0.0083
Guo M. et al., 2021 [31]	Cross-sectional observational	56 Chinese participants: 18 AD 20 MCI 18 NC	AD 2 M MCI 4 M NC 4 M	16S rRNA sequencing of fecal samples	MCI/AD	MMSE, MoCA	↓ Bacteroides, Lachnospira, Ruminiclostridium_9; ↑ Prevotella	Not reported	No intervention applied	*p <* 0.001
Liu P. et al., 2019 [32]	Cross-sectional prospective	97 Chinese participants: 32 NC 32 MCI 33 AD	NC 16 M MCI 14 M AD 19 M	16S rRNA sequencing of fecal samples	MCI/AD	MMSE, MoCA, CDR scores	↓ Firmicutes, Clostridiaceae, Ruminococcus, Lachnospira; ↑ Proteobacteria, Enterobacteriaceae, Escherichia/Shigella	The majority of healthy controls were patient’s spouses with the same diet	No intervention applied	*p =* 0.008
Pan et al., 2021 [33]	Case–control observational	22 MCI 26 NC	MCI 8 M NC 7 M	16S rRNA sequencing of fecal samples	MCI	MMSE. IADL	↑ Staphylococcus intermedius, S. lentus; ↓ Bacteroides salyersiae, B. gallinarum	Not reported	No intervention applied	*p =* 0.048

**Table 2:** Use of Prebiotics, Probiotics, Symbiotics and/or dietary modifications in patients with MCI and AD. LGG: Lactobacillus rhamnosus; MCI: mild cognitive impairment; AD: Alzheimer’s Dementia; CDG-2018: The 2018 Chinese Dietary Guidelines; rJDI12: revised Japanese Diet Index 12 items.

Study	Microbiota Intervention	Microbiota Changes			Marker
		Pre	Post	*p* Value	
Aljumaah et al., 2022 [13]	Daily supplementation with probiotic Lactobacillus rhamnosus GG (LGG) vs. placebo for 3 months	Prevotella ruminicola, Bacteroides thetaiotaomicron and Bacteroides xylanisolvens were increased in MCI	In the MCI group receiving LGG, there was a decrease in the abundance of Prevotella and Dehalobacterium	*p* = 0.0017	Microbiota biomarkers: Prevotella and Dehalobacterium
Zhang X. et al., 2021 [23]	Diet quality (CDG-2018): higher diet quality Lower diet quality	↓ Faecalibacterium, Roseburia	↑ Faecalibacterium, Roseburia ↑ Escherichia/Shigella	*p* < 0.005	microRNAs, dietary indices
Saji N. et al., 2022 [29]	Adherence to Japanese diet (rJDI12) Beneficial components: white rice, miso soup, fish and shellfish, green and yellow vegetables, seaweed, pickles, green tea, soybeans, fruit, mushrooms, coffee Less beneficial components: beef and pork	↓ SCFA-producers	↑Bifidobacterium, Clostridium butyricum and better cognition ↓ SCFA-producers	Participants without dementia consumed more fish and shellfish (*p* = 0.048), mushrooms (*p* = 0.015), soybeans and soybean-derived foods (*p* = 0.013), coffee (*p* = 0.024).	MMSE, dietary adherence scores

## Data Availability

No new data were created or analyzed in this study.
